# Single cell RNA and immune repertoire profiling of COVID-19 patients reveal novel neutralizing antibody

**DOI:** 10.1007/s13238-020-00807-6

**Published:** 2020-11-25

**Authors:** Fang Li, Meng Luo, Wenyang Zhou, Jinliang Li, Xiyun Jin, Zhaochun Xu, Liran Juan, Zheng Zhang, Yuou Li, Renqiang Liu, Yiqun Li, Chang Xu, Kexin Ma, Huimin Cao, Jingwei Wang, Pingping Wang, Zhigao Bu, Qinghua Jiang

**Affiliations:** 1 State Key Laboratory of Veterinary Biotechnology, Harbin Veterinary Research Institute, Chinese Academy of Agricultural Sciences, Harbin, 150001 China; 2 School of Life Science and Technology, Harbin Institute of Technology, Harbin, 150000 China; 3 Harbin Sixth Hospital, Harbin, 150000 China; 4 Key Laboratory of Biological Big Data (Harbin Institute of Technology), Ministry of Education, Harbin, 150001 China

There is no doubt that COVID-19 outbreak is currently the biggest public health threat, which has caused catastrophic consequences in many countries and regions. As host immunity is key to fighting against virus infection, it is important to characterize the immunologic changes in the COVID-19 patients, and to explore potential therapeutic candidates. The most efficient ways to end this pandemic are to vaccinate the susceptible population, and to use specific drugs, such as monoclonal antibodies against the viral spike protein (S protein), to treat the affected individuals. Several promising neutralizing antibodies have recently been reported (Cao et al., 2020; Lv et al., 2020). However, no antibody drug against COVID-19 has been approved yet in the world. Against the rapidly evolving SARS-CoV-2 virus, a cocktail of several non-competing antibodies may reach to the maximum treatment effect (Cai et al., 2020). Therefore, developing new potential antibodies remain be highly valuable.

Early-stage recovery patients maintain various immune responses and possess abundant protective antibodies in the circulation (Thevarajan et al., 2020). Therefore, we conducted a joint analysis using single cell transcriptome sequencing (scRNA-seq), single cell BCR sequencing (scBCR-seq) and deep BCR repertoire profiling to prioritize the therapeutically relevant neutralizing antibody sequences in patients who have recently cleared the virus.

Fresh blood samples were collected from a total of 16 COVID-19 patients at the time of hospital discharge (Table S1 and Supplementary Materials). PBMCs were divided into three aliquots for separate data generation: 1) single cell RNA sequencing, 2) single cell BCR V(D)J sequencing and 3) deep BCR repertoire sequencing (Fig. 1A and Table S1). In total, we obtained single cell 5′V(D)J sequencing data from 88,974 immune cells (Table S2) and immune receptor hypervariable regions from 6.9 million BCR clones (Table S3).

**Figure 1:**
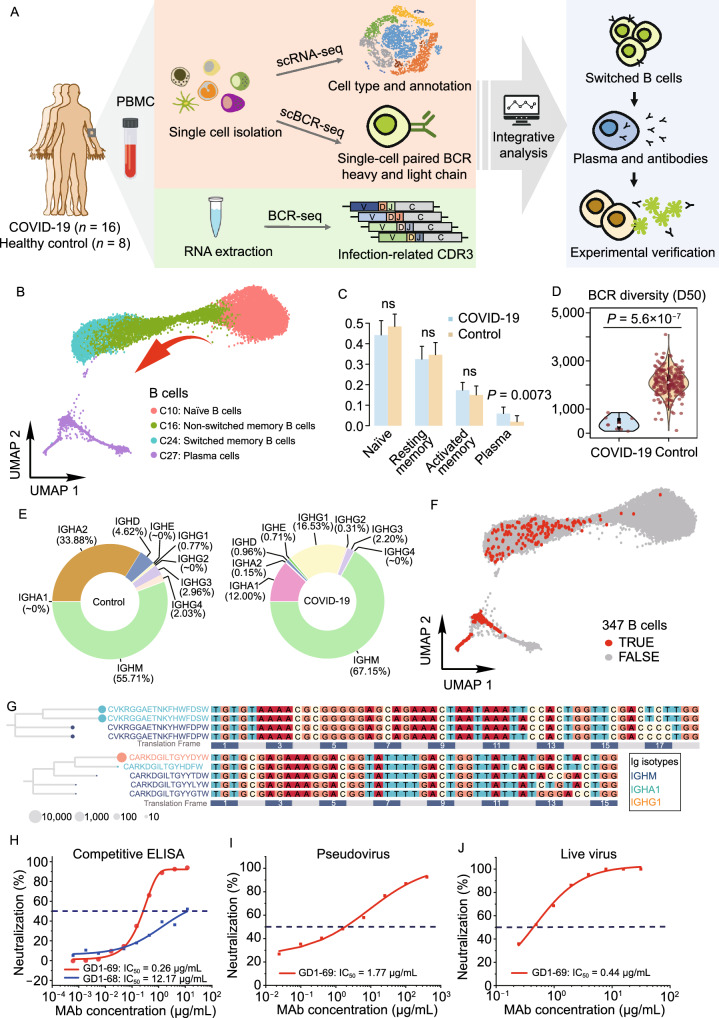
**Identification of clonally expanded BCR groups and neutralizing antibodies.** (A) Overview of experimental design. PBMC samples from recovered COVID-19 patients at discharge were collected and simultaneously performed single cell RNA-seq with 5′VDJ capture and deep B cell repertoire sequencing. (B) UMAP map of B cells from twelve COVID-19 patients and eight healthy controls, which formed a gradient of transcriptional states from naïve B cells to an activated memory B cells then to plasma cells. (C) Barplot showing the percentages for different B cell subgroups identified from single cell analysis, including naïve B cell (C10), resting memory B cells (C16), activated B cells (C24) and plasma cells (C27). Error bar labels one standard deviation of the data. Statistical significance was estimated using two-sided Wilcoxon rank sum test. ns, *P* > 0.05. (D) BCR diversity compared between patients and a control cohort of 235 deep BCR-seq samples. The diversity was measured using D50, which is proven robust to sequencing library size. *P* value was estimated using two-sided Wilcoxon test. (E) Pie chart showing the percentage of the 9 different Ig heavy chain isotypes of COVID-19 patients and healthy controls. Numbers in the parentheses are the averaged percentage of the corresponding Ig isotype across all the individuals, calculated using deep BCR-seq data. (F). UMAP plot shows the 347 potential antigen-specific BCRs are enriched in activated B cells (C24) and plasma cells (C27). (G) Lineage tree of selected BCRs heavy chain groups, with aligned DNA sequences as reference on the right. Different nucleotides are labeled with different colors, with translational frame marked beneath each plot. Each node represents a BCR clone, with color indicating the Ig isotype. The size of node reflects the frequency (in read counts) of the clone. (H) Competition binding to the COVID-19 virus RBD between antibody GD1-68 and GD1-69 and ACE2. X-axis represented the concentration of these two antibodies and Y-axis stood for percentage of uninterrupted RBD/ACE2 interaction. IC_50_, half-maximum inhibitory concentration. (I) The neutralization potency of GD1-69 was determined by pseudovirus-based neutralization assay. The mixtures of SARS-CoV-2 pseudovirus and serially diluted antibodies were added to HEK293T cells stably overexpressing human ACE2 (293T-ACE2 cells). IC50 values were calculated by fitting the cytopathic effect from serially diluted antibody to a sigmoidal dose-response curve. (J) The neutralization activity of the antibody GD1-69 was performed using a plaque reduction neutralization test assay. Serial dilutions of GD1-69 were incubated with SARS-CoV-2, and then added to pre-plated Vero E6 cell monolayers. The cells were incubated for 48 h with agarose overlay. Neutralizing titers (IC_50_ values) were calculated as the maximum antibody dilution yielding a 50% reduction in the number of plaques relative to that for control IgG protein

To reveal the changes of immune cells caused by SARS-CoV-2 infection, 8 healthy controls with single cell transcriptome data profiled by 10x Genomics were added in this study (Table S2 and Supplementary Materials). In total, we obtained 130,547 immune cells, comprising 88,974 cells (mean: 7,414 cells) for COVID-19 patients and 41,573 cells (mean: 5,196 cells) for healthy controls. Using unsupervised clustering, we found 28 distinct clusters representing different cell types (Fig. S1A), and identified the major PBMC cell types, including monocytes (C3, C4, C5, C6, C7, C12, C14, C19, C21, C22 clusters) with *CD14*, *CD68* and *CD163* expression; conventional CD4^+^ T cells (C1, C3, C8, C20 clusters); cytotoxic CD8^+^ T cells (C11, C15, C17, C18, C25, C26 clusters) which express *CD8*, *CCL5*, *NKG7*, *GNLY*; NK cells (C2 and C9 clusters) characterized by *KLRD1* (*CD94*), *KLRB1* (*CD161*), *NCR1* (*CD335*) expression; and B cells (C10, C16, C24 clusters) with *CD79*, *MS4A1* (*CD20*), *BANK1* expression (Fig. S1B). We also found 3 other clusters, annotated as regulatory CD4^+^ T cells (C23 cluster) with *FOXP3*, *CTLA4* (*CD152*) and *IL2RA* expression; plasma cells (C27 cluster) expressing *CD38* and *TNFRSF17* (*CD269*), and *CD1C*^+^*CD1E*^+^ dendritic cells (C28 cluster) (Fig. S1B). Cells were manually annotated by assessing the expression of classic marker genes and their expression similarity with purified bulk RNA-seq datasets.

Next, we focused our research on B cells (C10, C16, C24 and C27 clusters). We observed that B cells formed a gradient of transcriptional states from naïve B cells to an activated memory B cells then to plasma cells (Fig. 1B). We then compared the abundance of different B cell clusters between COVID-19 patients and controls, and observed average of 3.1 folds increase of plasma cells (C27 cluster) in the patients compared to healthy controls (Fig. 1C). In addition, we collected deep BCR-seq data from 235 additional healthy donors (Supplementary Materials). Compared to healthy individuals, COVID-19 patients showed significantly lower BCR diversity (Fig. 1D), indicating widespread B cell clonal expansions upon likely antigen recognition. With deep BCR sequencing, we investigated immunoglobulin (Ig) heavy chain isotypes for patients and controls. While in both cohorts, IgM presents the largest fraction in the peripheral repertoire, significantly higher abundance of IgG1 (*P* = 3.6 × 10^−7^, Wilcoxon test) and IgA1 (*P* < 2.2 × 10^−16^) antibodies were observed in COVID-19 patients (Fig. 1E). These changes are consistent with the anti-viral responses mounted by the adaptive immune system to clear viral particles in the blood and lung mucosa (Palladino et al., 1995). Antigen experienced B cells undergo somatic hypermutations (SHM) and Ig class switch recombination (CSR) to produce high-affinity antibodies and persistent protection (Gitlin et al., 2016).

We clustered similar heavy chain CDR3s to identify expanded B cell clonotypes, an approach proven effective to isolate antigen-specific antibodies (Hu et al., 2019). The screening process is shown in Fig. S2. We assembled a total of 74,634 BCR groups from all 16 COVID-19 patients, with each group representing a potential B cell clonal expansion event. Ig CSRs were determined based on the presence of different isotypes within each group. Consistently, we observed significantly enriched IgM to IgA1 or IgG1 events in most patients (Fig. S3). This analysis also allowed joint use of SHM and CSR to track the BCR evolution upon antigen stimulation. We identified a number of groups stemmed from naïve IgM B cells that sequentially acquired mutations in a focused region of the CDR3 loop (Zou et al., 2015), and ultimately evolved into IgA1 or IgG1 B cells (Figs. 1G and S4). The frequencies of the terminal clones are orders of magnitude than their ancestor IgM cells. In addition, when mapped to the scRNA-seq data, the expanded BCRs are enriched in activated B cells (C24) and plasma cells (Fig. 1F), corroborating their role in anti-viral responses.

Following the above results, we chose 347 BCR groups (Table S4) with highest potential to be antigen-specific using SHM and CSR as selection criteria (Supplementary Materials). In order to find potential high affinity antibodies, we kept the BCR heavy chain with the highest gene expression from single-cell BCR-seq in each BCR group, and then paired them with the corresponding light chain in single-cell BCR data to obtain a complete antibody sequence. Finally, we obtained 347 natural antibodies that were highly expanded in the patient's blood with evidence of antigen selection. In order to study the efficacy of these candidate antibodies, 100 antibodies were randomly selected for *in vitro* verification (Fig. S2 and Supplementary Materials). For each antibody, the DNA plasmids containing paired Ig heavy and light chains were synthesized, and transfected into HEK-293T cell line for antibody production. After purification, the antibodies were tested for the binding to the whole S protein, and the receptor-binding domain (RBD) of S protein using enzyme-linked immunosorbent assay (ELISA), respectively. 14 antibodies had strong binding to S protein (Table S5), and 3 of them (GD1-68, GD1-69 and GD1-75) had obvious binding to RBD, which may be neutralizing antibodies with clinical value. Among them, we found that GD1-69 has the highest binding affinity, with half-maximum inhibitory concentration IC_50_ = 0.26 μg/mL (Fig. 1H). The neutralization activity of the GD1-69 was further confirmed by pseudovirus-based neutralization assays with IC_50_ value of 1.77 μg/mL (Fig. 1I). To evaluate its neutralization potential against the authentic virus, we performed the plaque reduction neutralization test assay in Vero E6 cells using GD1-69 and live SARS-CoV-2 virus isolated from COVID-19 patients (Wang et al., 2020; Wu et al., 2020). GD1-69 showed viral inhibition with an IC_50_ of 0.44 μg/mL (Fig. 1J). Together, our data have shown that highly potent neutralizing monoclonal antibodies could be identified from early recovered patients by high-throughput single cell 5’V(D)J sequencing and bulk BCR repertoire sequencing.

So far, tens of millions of people have been infected by SARS-CoV-2, resulting in hundreds of thousands of deaths worldwide. SARS-CoV-2 neutralizing monoclonal antibodies are the most effective method for the treatment of infected patients. The joint use of scRNA-seq and deep repertoire profiling provided us the resolution of single cells, and also allowed us to acquire potential neutralizing antibodies. Using an ultrafast clustering algorithm, we uncovered over 74K expanding BCR groups out of 6 million sequences, each group bearing the evidence of recent antigen exposure and selection, i.e., somatic hypermutations and/or Ig class switch recombination. We identified 14 novel antibodies binding to S protein, and GD1-69 has the highest neutralizing activity. Currently there is a growing consensus that a combination of different, non-competing antibodies (Cai et al., 2020), or a “cocktail”, may reach to the optimum anti-viral effects, and our report had provided an additional promising reagent to the future medicines against the rapidly evolving SARS-CoV-2 virus.

Taken together, our study provides a valuable SARS-CoV-2 neutralizing monoclonal antibody named GD1-69. We anticipate future multidisciplinary efforts to carry forward the findings in our work to alleviate the ongoing crisis caused by the COVID-19 pandemic.

## Electronic supplementary material

Below is the link to the electronic supplementary material.

(PDF 1368 kb)

(XLSX 11 kb)

(XLSX 12 kb)

(XLSX 11 kb)

(XLSX 113 kb)

(XLSX 18 kb)
