# Supplementary material for: Single cell RNA and immune repertoire profiling of COVID-19 patients reveal novel neutralizing antibody
Source: Protein Cell. 2020 Nov 25;12(10):751–5. doi: 10.1007/s13238-020-00807-6 (PMC7686823; doi:10.1007/s13238-020-00807-6)
Supplement: 13238_2020_807_MOESM1_ESM — MATERIALS AND METHODS [file 13238_2020_807_moesm1_esm.pdf]

MATERIALS AND METHODS

Patient cohorts

COVID-19 patients were hospitalized at the Harbin sixth Hospital in China. Patients meeting the following criteria can be discharged: 1). Afebrile for >3 days, 2). Improved respiratory symptoms, 3). Pulmonary imaging shows obvious absorption of inflammation, 4). Nucleic acid tests negative for respiratory tract pathogen twice consecutively (sampling interval ≥ 24 hours). Fresh blood samples were collected from a total of 16 COVID-19 patients at the time of hospital discharge (**Table S1**). The clinical details were obtained from the Harbin sixth Hospital. Informed consent was obtained from the patients and this study was approved by the Ethics Committee in the Harbin sixth Hospital (Approval number: 2020NO.12).

Healthy control

In this study, eight healthy controls (HC1-HC8) were included in single cell RNA sequencing analysis (**Table S2**), among which HC1 and HC2 were from the official website of 10X genomics (https://support.10xgenomics.com/single-cell-vdj/datasets), HC3 is an in-house sample, which is PBMC sample collected from a healthy donor. The cryopreserved sample was purchased from AllCells.com. The sample was processed under standard protocol of 10x Genomics 5'V(D)J kit. HC4-HC8 (aged in their 50s to 80s) were download from Hashimoto et al (Hashimoto et al., 2019).

In this study, we included a healthy donor cohort of 235 individuals with BCR repertoire sequencing data available. The age of the donors ranges from 19 to 84, with median 42 years. Peripheral blood samples of all individuals were profiled using the iRepertoire platform. Notably, this platform uses cDNA reverse transcribed from mRNA of the B cell immunoglobulin heavy chain transcripts, the same as the BCR sequencing data produced in this work.

Single-cell 5' and V(D)J sequencing

Single-cell 5' and V(D)J sequencing was performed following the protocol provided by the 10X genomics Chromium Single Cell Immune Profiling Solution. Briefly, PBMCs cell suspensions (400-1000 living cells per microliter determined by CounterStar) were loaded on a Chromium Single Cell Controller (10x Genomics) to generate single-cell gel beads in emulsion (GEMs) by using Chromium Single Cell V(D)J Reagent Kits. Captured cells were lysed and the released RNA were barcoded through reverse transcription in individual GEMs. Each single-cell 5' and V(D)J libraries were sequenced on the Illumina's Novaseq 6000 using 150 paired-end reads.

Deep BCR repertoire sequencing

PBMCs were isolated by Ficoll-Paque (Paneco, Russia) density gradient centrifugation from fresh peripheral blood samples. RNA was isolated using the Trizol reagent (Invitrogen, USA) according to the manufacturer's protocol. Multiplex PCR amplification techniques were used to construct the sequencing libraries of BCR-H CDR3 regions, respectively. BCR repertoire sequencing were performed on Illumina sequencing platform by Capitalbio Technology

Corporation (Beijing, China). Raw BCR repertoires sequencing reads alignment and clonotype assembling were conducted by MiXCR (Bolotin et al., 2015).

Single-cell 5' V(D)J data processing

The analysis pipelines in Cell Ranger (10X Genomics, version 3.1.0) were used for single cell sequencing data processing. Gene expression matrix files were generated using cellranger count with –transcriptome = refdata-cellranger-GRCh38-3.0.0 for each sample. V(D)J sequence assembly and paired clonotype calling were performed using cellranger vdj with –reference = refdata-cellranger-vdj-GRCh38-alts-ensembl-3.1.0 for each sample. Outputs of cellranger count for individual samples were integrated using cellranger aggr with --normalize = none. Raw counts for each individual were normalized by scaling to the total number of UMI, per cell, and log transformed by NormalizeData function in R package Seurat (version: 3.0.2) (Butler et al., 2018; Stuart et al., 2019). Batch effects were removed and data sets from each individual were integrated by the standard Seurat v3 integration workflow (Stuart et al., 2019). Cell-cycle scoring was done by CellCycleScoring function in Seurat. Then cell cycle scores were regressed during data scaling by ScaleData function in Seurat. Normalized distributions were subsequently scaled to have 0 mean, and standard deviation of 1.

Principal component analysis was computed based on the scaled expression values of variable genes. First 30 principal components were used to construct a KNN graph using FindNeighbors function in Seurat, the edge weights between any two cells based on the shared overlap in their local neighborhoods (Jaccard similarity). To cluster the cells, Louvain algorithm was used to iteratively group cells together, with the goal of optimizing the standard modularity function. First 20 principal components were used to generate tSNE projections. Cell types were annotated by manually checking the results from CIBERSORT (Newman et al., 2019) and SingleR (Aran et al., 2019), which leverages reference transcriptomic datasets of pure cell types to infer the cell of origin of each single cell independently. Red blood cells, platelets, doublet cells, and other artifacts were removed from the downstream analysis.

iSMART groups analysis

For each repertoire, we extracted CDR3s with more than 5 reads, to include effector/memory B cell population at maximum. As a result, each sample provided 85K to 370K sequences. We processed the BCR clustering using iSMART (Zhang et al., 2020) for each patient separately. This is based on the previous observation that inter-patient BCR sharing is negligible compared to inter-patient TCR sharing (Hu et al., 2019). We then combined iSMART results from all patients and obtained 74,634 BCR groups. To select the groups that are more likely to be antigen-specific, and suitable for downstream modeling (Figure S2), we imposed the following selection criteria: 1) There are at least five distinct CDR3 nucleotide sequences in the group; 2) The group must have evidence of IgM to IgG or IgM to IgA class switch recombination; 3) At least one CDR3 in the group must be found in the single cell data, to acquire paired light chain information. After selection, there are 347 remaining BCR groups, and we reported the ones with highest clonal abundance within each group.

Reconstruction of BCR lineage tree

The input data, grouped BCRs (heavy chains only), were generated by iSMART (Zhang et al., 2020) from BCR sequences mixed from BCR-Seq and 10x Genomics Immune Profiling in each sample. Clustal Omega (Sievers et al., 2011) was used to perform multiple sequence alignment (MSA) of BCR nucleic acid sequences in each BCR group. Using model GY94, Igphyml (Hoehn et al., 2017) successfully generated phylogenetic trees from the results of multiple sequence alignment. Ete3 (Huerta-Cepas et al., 2016) was used to conduct visualization and filtration on the phylogenetic trees.

Analysis of BCR class-switch recombination

The class switch (CS) took BCR groups generated by iSMART (Zhang et al., 2020) as input. For each sample, we calculated the number of BCR groups shared between any two isotypes. Considering the biological order of CS events in immunological process, we filtered out the pair of IGHA1 and IGHA2 and adjusted the CS direction of each pairs of isotypes to the right order, remaining 15 CS events in the end (IGHM -> IGHG3/ IGHG1 /IGHG2/ IGHA1/ IGHA2, IGHG3 -> IGHG1/ IGHG2/ IGHA1/ IGHA2, IGHG1 -> IGHG2/ IGHA1/ IGHA2, IGHG2 -> IGHA1/ IGHA2). For each sample, we used Fisher's exact test to identify significantly enriched CS events, and the p values were corrected using the Benjamini-Hochberg method.

The measure of BCR diversity

We used D50 value to estimate the BCR diversity for each sample. We decreasingly ordered the clone counts for top 10,000 clonotypes with highest clonal frequency. The D50 value was defined as:

$$D50^j = argmin_x(\sum_{i=1}^{x} N_i^j - \frac{1}{2}\sum_{i=1}^{Thr} N_i^j > 0)$$

Where the Thr=10,000, N_i^j is the clone count of the i th BCR clonotype (i ≤ Thr) in sample j.

Screening for neutralizing antibodies

The candidate antibody sequences were screened from 347 BCR group (**Figure S2**). We selected 347 BCR heavy chains in each group according to the following conditions: 1) The sequence came from single cell BCR data, since it can provide enough information about paired chains and V(D)J segments to complete antibody assembly; 2) Keep the heavy chain with the highest expression. Finally, 347 natural antibody sequences were obtained by pairing 347 heavy chains with their corresponding light chains in single cell BCR data.

Monoclonal antibody expression and purification

Paired heavy chain and light chain cDNA were selected for codon optimization and cloned into the expression vector containing the constant region of human IgG1. HEK293 cells were transfected with the equal amounts of heavy chain and light chain plasmids to express IgG

monoclonal antibodies. The protein supernatant was purified using affinity chromatography to obtain recombinant monoclonal antibodies.

ELISA quantification

The enzyme-linked immunosorbent assay (ELISA) plates were coated with 0.01 μg/ml and 5 μg/ml SARS-CoV-2 S protein, as well as 0.01 μg/ml and 1 μg/ml SARS-CoV-2 RBD region in phosphate-buffered saline (PBS) at 4 ℃ overnight. After standard washing and blocking, 1 μg/ml antibodies were added to each well and incubated at room temperature for 2 hours. The plates were washed and incubated with 0.08 μg/ml goat anti-human IgG (H+L)/HRP (JACKSON) for 1 h at room temperature. Using chromogen solution as the substrate, the absorbance at 450 nm was measured by a microplate reader.

For the competition ELISA, Biotinylated RBD (0.3 μg/ml) was immobilized on the ELISA plates. After standard washing, 0.05 μg/ml ACE2-His was added to the wells, and then the diluted antibodies were added immediately. After incubation at room temperature for 2 hours, the plate was washed and anti-His/HRP (1:3000) was added. After incubation at room temperature for 1 hour, the absorbance at 450 nm was measured by a microplate reader. Compared with negative control, the inhibition rate of ACE2/RBD binding was calculated.

Pseudovirus neutralization assay

The human ACE2 overexpression stable HEK293T cells (293T-ACE2 cells) (2×10^4 cells/well) were seeded in 96-well plates. For the neutralization assay, 50 μl SARS-CoV-2 pseudoviruses (~2×10^4 RLU) were incubated with serial dilutions of antibodies for 1 h at 37 ℃, then added to the 96-well 293T-ACE2 cells. Luciferase luminescence value was detected by chemiluminescence method, and the pseudovirus neutralization inhibition rate of the antibody was calculated according to the luciferase luminescence value to evaluate the neutralization effect of the antibody.

SARS-CoV-2 neutralization assay

All experiments with infectious SARS-CoV-2 were performed in the biosafety level 4 facilities in the Harbin Veterinary Research Institute (HVRI) of the Chinese Academy of Agricultural Sciences (CAAS), which is approved for such use by the Ministry of Agriculture and Rural Affairs of China. The neutralizing activity of the antibody GD1-69 was assessed using a plaque reduction neutralization test assay as described previously (Wu et al., 2020). Briefly, serial dilutions of GD1-69 with DMEM medium were incubated with 50 PFU of the SARS-CoV-2 at 37 ℃ for 1 h, and then added to pre-plated Vero E6 cell monolayers in 24-well plates. The plates were incubated in CO2 incubator at 37 ℃ for 48 h with agarose overlay. Neutralizing titer was calculated as the maximum antibody dilution yielding a 50% reduction in the number of plaques relative to that for control IgG protein.

Statistical Analysis

All statistical analyses and visualization were performed using R the statistical programming

language (version 3.6.1). Two sample tests were performed using two-sided Wilcoxon rank sum test. If multiple tests were performed for a single analysis, we used BH procedure to correct for FDR. For all the boxplots displayed in the figures, the middle line defines the median value, with borders of the boxes indicating the 25% (Q1) and 75% (Q3) quartiles of the data. Lower and upper whiskers corresponded to Q1-1.5IQR and Q3+1.5IQR, where IQR is short for inter-quartile range.

References:

Aran, D., Looney, A.P., Liu, L., Wu, E., Fong, V., Hsu, A., Chak, S., Naikawadi, R.P., Wolters, P.J., Abate, A.R., *et al.* (2019). Reference-based analysis of lung single-cell sequencing reveals a transitional profibrotic macrophage. Nature Immunology 20, 163-172.

Bolotin, D.A., Poslavsky, S., Mitrophanov, I., Shugay, M., Mamedov, I.Z., Putintseva, E.V., and Chudakov, D.M. (2015). MiXCR: software for comprehensive adaptive immunity profiling. Nature Methods 12, 380-381.

Butler, A., Hoffman, P., Smibert, P., Papalexi, E., and Satija, R. (2018). Integrating single-cell transcriptomic data across different conditions, technologies, and species. Nature Biotechnology 36, 411-420.

Hashimoto, K., Kouno, T., Ikawa, T., Hayatsu, N., Miyajima, Y., Yabukami, H., Terooatea, T., Sasaki, T., Suzuki, T., Valentine, M., *et al.* (2019). Single-cell transcriptomics reveals expansion of cytotoxic CD4 T cells in supercentenarians. Proc Natl Acad Sci U S A 116, 24242-24251.

Hoehn, K.B., Lunter, G., and Pybus, O.G. (2017). A Phylogenetic Codon Substitution Model for Antibody Lineages. Genetics 206, 417-427.

Hu, X., Zhang, J., Wang, J., Fu, J., Li, T., Zheng, X., Wang, B., Gu, S., Jiang, P., Fan, J., *et al.* (2019). Landscape of B cell immunity and related immune evasion in human cancers. Nature Genetics 51, 560-567.

Huerta-Cepas, J., Serra, F., and Bork, P. (2016). ETE 3: Reconstruction, Analysis, and Visualization of Phylogenomic Data. Molecular Biology and Evolution 33, 1635-1638.

Newman, A.M., Steen, C.B., Liu, C.L., Gentles, A.J., Chaudhuri, A.A., Scherer, F., Khodadoust, M.S., Esfahani, M.S., Luca, B.A., Steiner, D., *et al.* (2019). Determining cell type abundance and expression from bulk tissues with digital cytometry. Nature Biotechnology 37, 773-782.

Sievers, F., Wilm, A., Dineen, D., Gibson, T.J., Karplus, K., Li, W., Lopez, R., McWilliam, H., Remmert, M., Söding, J., *et al.* (2011). Fast, scalable generation of high-quality protein multiple sequence alignments using Clustal Omega. Mol Syst Biol 7, 539.

Stuart, T., Butler, A., Hoffman, P., Hafemeister, C., Papalexi, E., Mauck, W.M., III, Hao, Y., Stoeckius, M., Smibert, P., and Satija, R. (2019). Comprehensive Integration of Single-Cell Data. Cell 177, 1888-1902.e1821.

Wu, S., Zhong, G., Zhang, J., Shuai, L., Zhang, Z., Wen, Z., Wang, B., Zhao, Z., Song, X., Chen, Y., *et al.* (2020). A single dose of an adenovirus-vectored vaccine provides protection against SARS-CoV-2 challenge. Nat Commun 11, 4081.

Zhang, H., Liu, L., Zhang, J., Chen, J., Ye, J., Shukla, S., Qiao, J., Zhan, X., Chen, H., Wu, C.J., *et al.* (2020). Investigation of Antigen-Specific T-Cell Receptor Clusters in Human Cancers. Clin Cancer Res 26, 1359-1371.

Supplementary Figures

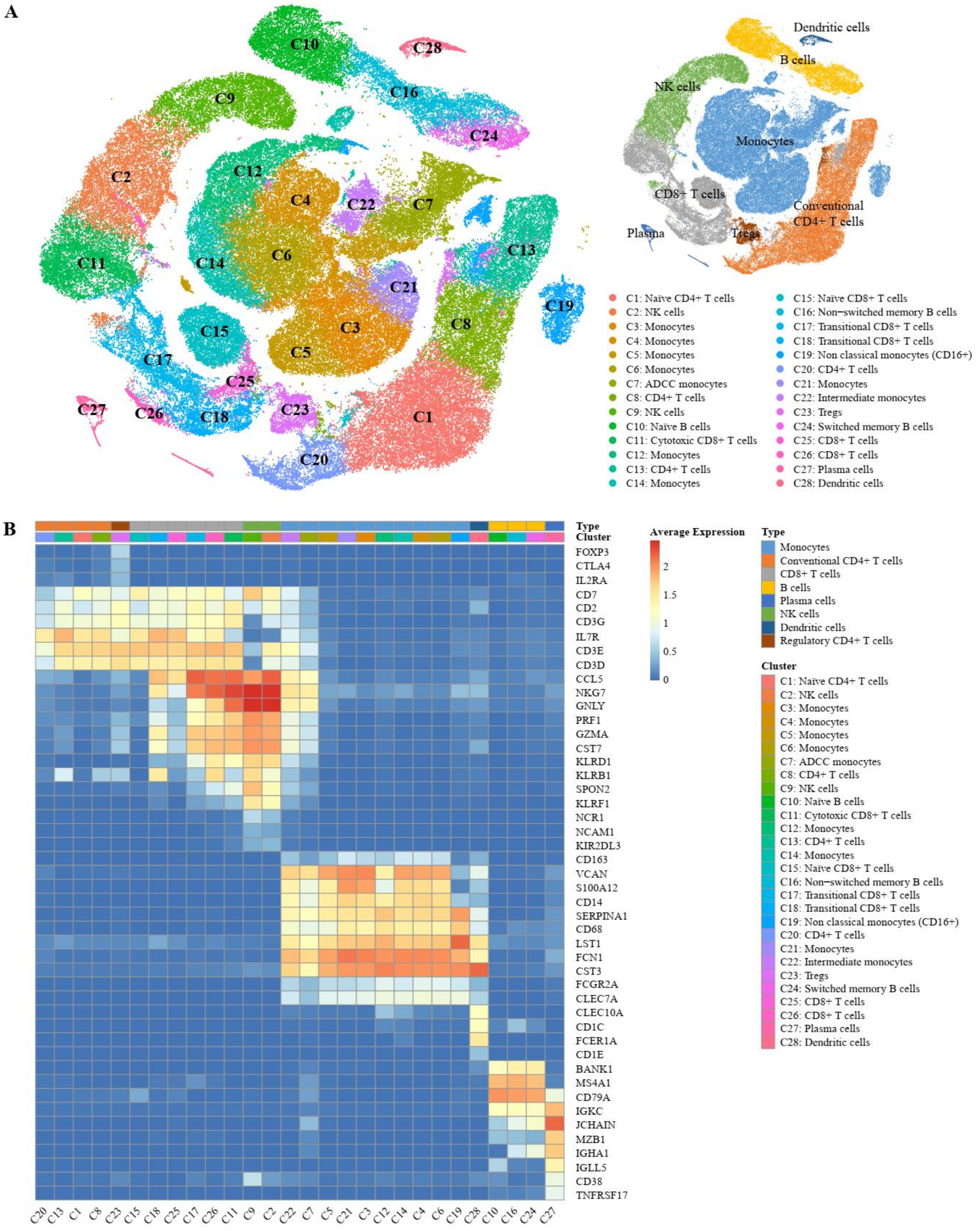

Figure S1. Single-cell transcriptome profiling of PBMCs of COVID-19 patients and healthy controls. (**A**) tSNE map of PBMCs from twelve COVID-19 patients and eight healthy controls, with numerically labeled cluster numbers (bigger panel) or major PBMC cell subtypes (smaller panel). Clustering was based on unsupervised k-means using the normalized gene expression values after batch effect removal (**B**) Heatmap showing the average log-normalized gene

expression of marker genes for all eight cell types discussed in the main text. For normalization, gene counts for each cell were divided by the total counts for that cell and multiplied by the scale.factor (1e6), and followed by log transformation. Markers were ordered to visualize the differences between cell types.

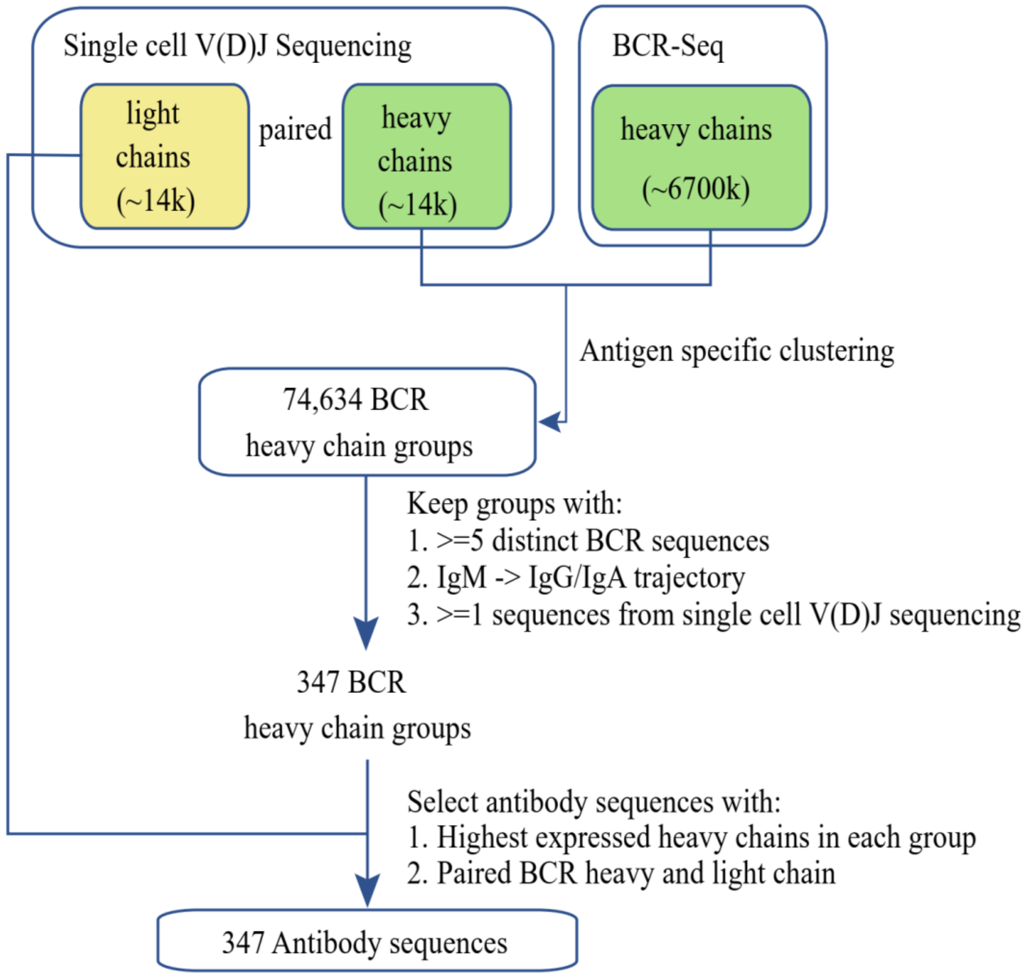

Figure S2. Screening process of COVID-19 antigen-specific neutralization antibody candidates. We clustered similar heavy chain CDR3s to identify expanded B cell clonotypes from single cell BCR sequencing data and deep BCR sequencing data. A total of 74,634 BCR groups were assembled from all 16 COVID-19 patients, with each group representing a potential B cell clonal expansion event. Ig CSRs were determined based on the presence of different isotypes within each group. We chose total 347 BCR heavy chains sequenced by single cell V(D)J sequencing with highest expression in each group. For these BCR heavy chains, we paired them with corresponding light chains from single cell BCR data to get the antibodies sequences for further validation assays.

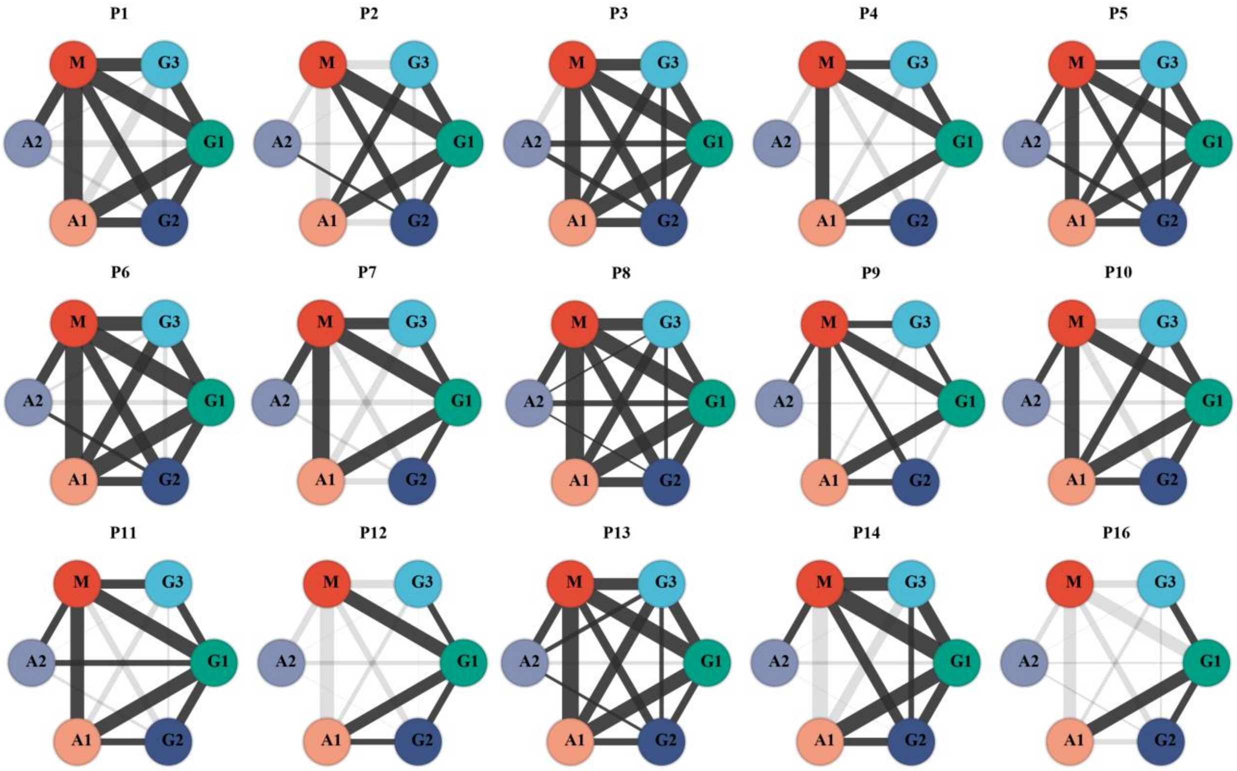

Figure S3. Visualization of Ig isotype co-occurrence within B cell clusters by samples. Lines connecting two nodes indicate the sharing between two corresponding Ig subclasses, with darker color for higher enrichment level by fisher-exact test and wider width for higher sharing count. The enrichment level is the ratio of observed and expected switches if assuming Ig isotypes are independently distributed among B cell clusters.

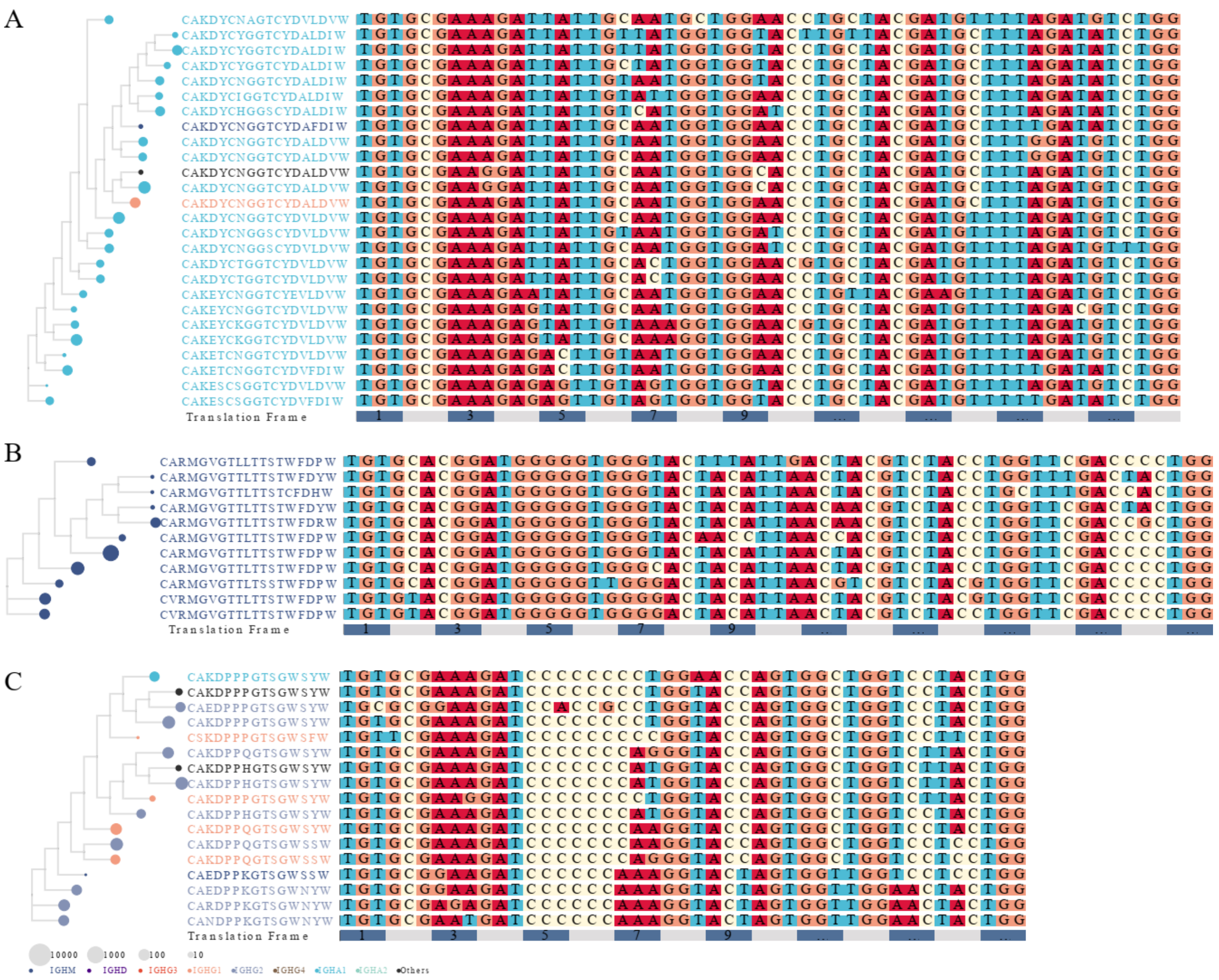

Figure S4. Lineage tree of BCRs heavy chains group 140 from patient P5 (A), group 6 from patient P1 (B) and group 94 from patient P11 (C), generated by iSMART with aligned cDNA sequences on the right. The sequences were mixed from BCR-seq and 10x Genomics Immune Profiling and only heavy chains were remained for analysis. Each node represents a BCR heavy chain, colored by corresponding isotype. The size of node represents BCR frequency in the group.

Supplementary tables

Table S1. Data information of 16 COVID-19 patients

Table S2. The summary of single cell RNA sequencing data

Table S3. The summary of BCR-seq data

Table S4. The filtered 347 BCRs of BCR repertoire analysis.

Table S5. The result of ELISA test of 100 antibody candidates.
